# Protein carbonylation in food and nutrition: a concise update

**DOI:** 10.1007/s00726-021-03085-6

**Published:** 2021-10-20

**Authors:** Mario Estévez, Silvia Díaz-Velasco, Remigio Martínez

**Affiliations:** Food Technology, IPROCAR Research Institute, Universidad de Extremadura, 10003 Cáceres, Spain

**Keywords:** Protein carbonylation, *α*-Aminoadipic semialdehyde, *α*-Aminoadipic acid, Oxidative stress, Maillard reaction, Protein oxidation, Nutrition, Safety, Disease

## Abstract

Protein oxidation is a topic of indisputable scientific interest given the impact of oxidized proteins on food quality and safety. Carbonylation is regarded as one of the most notable post-translational modifications in proteins and yet, this reaction and its consequences are poorly understood. From a mechanistic perspective, primary protein carbonyls (i.e. *α*-aminoadipic and *γ*-glutamic semialdehydes) have been linked to radical-mediated oxidative stress, but recent studies emphasize the role alternative carbonylation pathways linked to the Maillard reaction. Secondary protein carbonyls are introduced in proteins via covalent linkage of lipid carbonyls (i.e. protein-bound malondialdehyde). The high reactivity of protein carbonyls in foods and other biological systems indicates the intricate chemistry of these species and urges further research to provide insight into these molecular mechanisms and pathways. In particular, protein carbonyls are involved in the formation of aberrant and dysfunctional protein aggregates, undergo further oxidation to yield carboxylic acids of biological relevance and establish interactions with other biomolecules such as oxidizing lipids and phytochemicals. From a methodological perspective, the routine dinitrophenylhydrazine (DNPH) method is criticized not only for the lack of accuracy and consistency but also authors typically perform a poor interpretation of DNPH results, which leads to misleading conclusions. From a practical perspective, the biological relevance of protein carbonyls in the field of food science and nutrition is still a topic of debate. Though the implication of carbonylation on impaired protein functionality and poor protein digestibility is generally recognized, the underlying mechanism of such connections requires further clarification. From a medical perspective, protein carbonyls are highlighted as markers of protein oxidation, oxidative stress and disease. Yet, the specific role of specific protein carbonyls in the onset of particular biological impairments needs further investigations. Recent studies indicates that regardless of the origin (in vivo or dietary) protein carbonyls may act as signalling molecules which activate not only the endogenous antioxidant defences but also implicate the immune system. The present paper concisely reviews the most recent advances in this topic to identify, when applicable, potential fields of interest for future studies.

## Introduction

Oxidative stress is a major cause of post-translational changes in proteins and includes severe chemical modifications such as aggregation via protein crosslinks and fragmentations via peptide scission (Davies 2016; Kehm et al. 2021). The oxidation of the aminoacids’ side chain leads to the formation of derivatives of assorted nature including sulphur compounds (i.e. methionine sulphoxide; from methionine), aromatic species (i.e. kynurenines; from tryptophan) and carboxylic acids (*α*-aminoadipic acid; *α*-AA; from lysine) (Estévez et al. 2020; Davies 2016). In food systems, these chemical changes are manifested in loss of protein functionality and digestibility with relevant consequences in terms of food protein quality and nutritional value (Soladoye et al. 2015; Xiong and Guo 2021). In cells and living organisms, protein oxidation is linked to biological impairments, aging and the onset of various pathological conditions (Akagawa 2020).

The formation of protein carbonyls typically responds to the oxidative deamination of alkaline amino acids such as lysine, arginine and proline (Estévez 2011; Davies 2016). The *α*-aminoadipic semialdehyde (*α*-AS) is a specific oxidation product from lysine and account for up to the 70% of total protein carbonyls in cells (Requena et al. 2001) and food systems (Estévez 2011). Owing to their ubiquity in oxidized proteins and their simple detection and quantification by the routine dinitrophenylhydrazine (DNPH) method, protein carbonyls have been emphasized as general markers of protein oxidation in foods, cells and tissues (Estévez 2011; Hellwig 2020). Yet, limitations in both, accuracy of the technique, and the interpretation of the data, makes the DNPH method unsuitable to gain knowledge of scientific in-depth and mechanistic nature (Estévez et al. 2019; Hellwig 2020; Estévez 2021). The understanding of the intricate chemistry behind protein carbonylation is essential to comprehend its biological meaning. More accurate and advanced methodologies have been proposed to gain further insight into the molecular basis of the formation and biological effects of protein carbonyls (Fedorova et al. 2014; Hellwig 2020). Owing to the application of spectroscopic and mass spectrometric methodologies, recent relevant advances have been made in regards to the role of the Maillard reaction and the glyco-oxidation as alternative routes for protein carbonylation in biological samples (Trnková et al. 2015; Arcanjo et al. 2018; Luna et al. 2021). On the same line, recent studies have reported innovative evidence on the toxicological effects of dietary protein carbonyls and other oxidized amino acids (Estévez and Xiong 2019) and the role of these compounds in cell signalling mechanisms (Wong et al. 2012; Kehm et al. 2021). This review concisely collects and analyses the most recent advances in this topic and aims to elucidate future challenges.

## Concise update on carbonylation chemistry

**Fig. 1:**
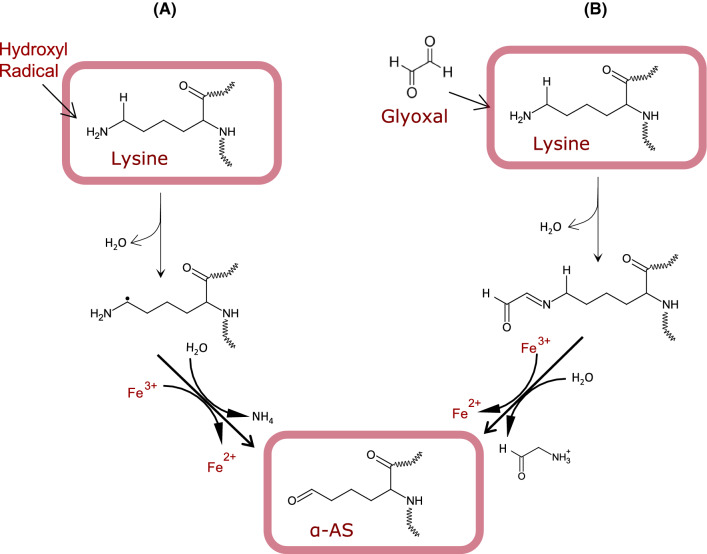
Mechanisms of primary carbonylation: formation of α-aminoadipic acid (*α*-AS). **A** Radical-mediated oxidative deamination of protein-bound lysine; **B** Maillard-mediated oxidative deamination of protein-bound lysine

Carbonylation is an irreversible post-translational modification through which carbonyl moieties are formed and/or introduced in proteins. This reaction takes place under various mechanisms of diverse nature though it is generally accepted that carbonylation typically takes place in a pro-oxidative environment and as a result of oxidative stress (Fedorova et al. 2014; Akagawa 2020). Notable exceptions should be denoted such as the carbonylation that occurs in certain cells and tissues under physiological conditions such as the formation of *α*-AS from lysine prior to condensation reactions in connective tissue (Xu and Shi 2014). This reaction, is yet, enzymatically controlled (e.g. lysyl oxidase) and does not respond to the non-enzymatic oxidative stress-induced conditions in which uncontrolled protein carbonylation commonly occurs. Depending on whether carbonyls are formed in proteins or introduced in proteins subsequent to its formation, two types of protein carbonylation can be clearly discriminated. “Primary carbonyls” are formed on-site consequently to an oxidative damage to the protein structure while “secondary protein carbonyls” are formed from lipid oxidation and subsequently introduced in proteins via covalent linkages (Estévez et al. 2019; Akagawa 2020). In turn, the former can be generated via three mechanisms, namely, (i) oxidative deamination of alkaline amino acids (lysine, threonine, arginine and proline) by a radical-mediated mechanism; (ii) oxidative deamination of alkaline amino acids by dicarbonyls from the Maillard reaction (MR); and (iii) by oxidative cleavage of the peptide backbone via the *α*-amidation pathway or via oxidation of glutamyl side chains (Berlett and Stadtman 1997; Estévez 2011; Akagawa 2020). Quantitatively, it is generally assumed that the latter among these three mechanisms is of negligible importance, and it is unusually reported as a source of protein carbonyls in biological samples. Among the relevant primary carbonylation mechanisms, the radical-mediated, known as ‘Stadtman’ pathway (Fig. 1A), normally involves a metal-catalysed oxidation (MCO) mechanism with the Fenton reaction being source of hydroxyl radicals and other reactive oxygen species (ROS) (Stadtman and Levine 2003). The reactive species would attack the *ε*-amino moiety from the alkaline amino acid by abstracting a hydrogen atom from the neighbouring carbon, leading to the formation, in a first step, of an imino group. This intermediate and unstable product is readily hydrolysed to form the corresponding protein carbonyl. According to this reaction pathway, the *α*-AS, also known as allysine, is formed from the oxidative deamination of lysine while the *γ*-glutamic semialdehyde (*γ*-GS) is formed from arginine and proline (Stadtman and Levine 2003; Estévez 2011). The alternative MR mediated mechanism, known as the ‘Suyama’ pathway (Fig. 1B), and also described as glyco-oxidation, implies the degradation of reducing sugars and the formation of reactive *α*-dicarbonyls such as glyoxal (GO) and methylglyoxal (MGO) (Akagawa et al. 2005). These highly reactive dicarbonyls react with *ε*-amino groups in proteins causing the oxidative deamination of the alkaline amino acids and the formation of the same aforementioned protein carbonyls. Hence, both mechanisms lead to the same oxidation products and depending on the experimental approach and detection method, the relative contribution of each pathway to the carbonylation of proteins is indefinite. In food systems, most authors assume that protein oxidation, typically assessed as protein carbonylation, is the result of a radical-mediated mechanism (Estévez 2011; Hellwig 2020). Yet, recent results indicate that the glyco-oxidation of proteins leading to the formation of primary carbonyls is not only applicable. Under favourable conditions (e.g. occurrence of reducing sugars), this mechanism may be even most relevant than ROS-mediated carbonylation (Luna and Estévez 2019; Luna et al. 2021). Villaverde and Estévez (2013) originally reported the occurrence of Maillard-mediated protein carbonylation in myofibrillar proteins at as low concentrations of glucose as those found in post-mortem muscle (~ 0.02 M). Luna and Estévez (2018) subsequently observed that glucose (0.05 M) was more efficient than hydrogen peroxide (0.6 mM) in reacting with metal ions to create the required pro-glyco-oxidative environment to induce protein carbonylation in a variety of food proteins, including meat proteins, ovalbumin, *β*-lactoglobulin and soy proteins. In a following study, the same authors confirmed the involvement of GO and MGO in the “Suyama pathway” and found that glyco-oxidized meat and dairy proteins displayed impaired functionality and digestibility (Luna and Estévez 2019). Recent studies performed in a variety of food proteins such as glutenin (Wang et al. 2019a) and *α*-lactalbumin (Wu et al. 2021), have confirmed severe chemical changes induced by reactive Maillard dicarbonyls. While this mechanism may be highly relevant in food systems with high protein and sugar concentration (i.e. fermented meat products or certain dairy foods), the carbonylation of proteins in such foods is rarely attributed to this pathway by food scientists. The carbonylation of proteins during digestion has also been hypothesized to be affected by sugar concentration (Duque-Estrada et al. 2019). In a recent study, we observed that 10 mg/mL of glucose and GO strikingly promoted the carbonylation of meat proteins during simulated gastric digestion (unpublished data). The concentration of total protein carbonyls in the glyco-oxidized proteins increased ~ 20-fold times in meat proteins digested in the presence of glucose vs the control counterparts.

In living systems, the “Suyama pathway” was originally described to occur in plasma proteins from diabetic rats (Akagawa et al. 2006). The authors precisely described the underlying mechanisms and the role of GO and MGO in such carbonylation process (Akagawa et al. 2006). In the last five years, subsequent studies have contributed to identify the routes and mechanisms of protein carbonylation in proteins from plasma and other tissues under pathological hyperglycemic conditions. Given the connection between protein carbonylation in diabetic patients and the onset of oxidative stress and the metabolic syndrome (Hecker and Wagner 2018), strategies of carbonylation inhibition have been proposed to alleviate the associated biological impairments. Özyurt et al. (2016) reported fourfold increases of specific semialdehydes in human hemoglobin and human serum albumin (HSA) upon exposure to simulated hyperglycemic conditions (12 mM glucose/0.2 mM Fe^3+^) for 10 days. Epicatechin, epigallocatechin and epigallocatechin-3-gallate at 0.7 μM, were found to keep protein carbonyls at basal levels through anti-glycation mechanisms. Arcanjo et al. (2018) was able to double the concentration of *α*-AS and *γ*-GS in HSA upon incubation with pathological concentrations of GO and MGO (0.4 mM; Lapolla et al. 2003) for 48 h. The same authors found that resveratrol (2.5 μM) effectively inhibited the carbonylation of HSA by forming adducts with the aforementioned Maillard dicarbonyls. More recently, Luna et al. (2021) reported that glucose induces dose-dependent carbonylation of HSA at relevant pathophysiological concentrations (4–12 mM) and observed that the main protein carbonyl (*α*-AS) suffered a further oxidation step to yield the lysine oxidation end product, the *α*-amino adipic acid (*α*-AA). Interestingly, this oxidized form of lysine has been recently emphasized as an early and reliable marker of insulin resistance and diabetes in humans (Lee et al. 2019).

**Fig. 2:**
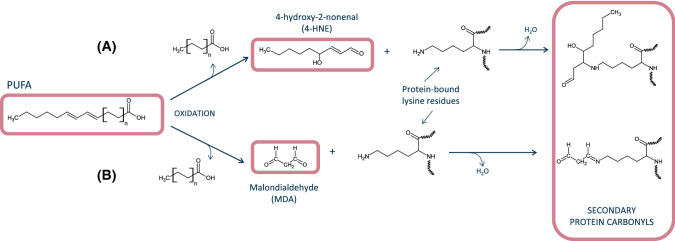
Mechanisms of secondary carbonylation. **A** Michael addition of 4-hydroxynonenal to protein-bound lysine; **B** Michael addition of malondialdehyde to protein-bound lysine

Finally, considerable progresses have also been made in relation to the understanding of the chemistry involved in the formation of secondary protein carbonyls. Malondialdehyde (MDA), 4-hydroxynonenal (4-HNE) and other lipid-derived carbonyls have been found to react with protein-bound amino groups and form complexes with food proteins (Zhou et al. 2015; Gürbüz and Heinonen 2015; Wang et al. 2019b). Unlike the aforementioned dicarbonyls from MR (GO and MGO), MDA and other lipid carbonyls are unable to induce the oxidative deamination of lysine residues to yield primary protein carbonyls such as *α*-AS from lysine. Instead, MDA and 4-HNE remain bound to proteins as secondary protein carbonyls via a Michael addition-type reaction (Fig. 2). This reaction occurs between MDA [and its degradation products, acetaldehyde (AA) and formaldehyde (FA) and other carbonyls], and lysine and other alkaline amino acids to form adducts of assorted stability. It is worth noting that a second aldehyde moiety from the added MDA remains free and available to react with DNPH or other derivatization agents, which enables its detection as (secondary) protein carbonyls. Alternatively, the free aldehyde from a protein-bound MDA could react with another lysine residue, which leads to the formation of intra- and/or intermolecular covalent crosslinks. In a recent study, Estévez et al. (2019) observed that the accretion of protein hydrazones (DNPH-derivatized carbonyls) in HSA was affected by the exposure to a broad concentration range of MDA (0.05–5 mM). The authors concluded that the concentration of secondary protein carbonyls, corresponding to protein-bound MDA, increased with the concentration of MDA. The extent of carbonylation in HSA exposed to 5 mM peaked at around 22 nmol per mg of protein. At such experimental concentrations, the addition of MDA molecules to lysine residues occurs at so high levels, that the formation of primary protein carbonyls from such alkaline amino acid is completely inhibited. The authors report that in foods or biological systems subjected from moderate to severe lipid oxidation (concentrations of MDA between 0.05 and 1 mM), between 50 and 80% of protein carbonyls detected by the DNPH method would be secondary carbonyls. According to the recent and interesting results reported by Wang et al. (2019b), MDA may not only be responsible of secondary carbonylation of myoglobin and myofibrillar proteins from rabbit skeletal muscle. This lipid-derived carbonyl may be able to induce the formation of hypervalent myoglobin species, which promotes the creation of a pro-oxidative environment in the muscle tissue, and worsen, in turn, the oxidative stress. The comprehensive review by Tsikas (2017) provides further details on the formation, reactivity and analytical methods of MDA. Other reactive carbonyls such as acrolein and crotonaldehyde have been found to induce carbonylation stress in cells and other biological systems (Mello et al. 2007). Their implication in the secondary carbonylation of food proteins requires further elucidation.

## Concise update on methodological approaches

The DNPH method is the most common procedure for the routine quantification of total protein carbonyls from a biological sample and the results are broadly used as a general index of protein oxidation (Estévez 2011; Hellwig 2020). The technique involves a concurrent spectrophotometric determination of DNPH-derivatized protein-bound carbonyls [as 2,4-dinitrophenyl (DNP) hydrazones] and the total protein content of the sample (Oliver et al. 1987). Eventually, the results are expressed as nmol protein hydrazones per mg of protein. It is worth emphasizing that (i) the original procedure was set to evaluate protein oxidation in plasma samples (Oliver et al. 1987) and that (ii) the DNPH reacts with carbonyl moieties regardless of the origin or formation pathway. Because of this, the method has been modified to facilitate the analysis of proteins from solid samples and low solubility (i.e. myofibrillar proteins) and to avoid interferences caused by lipid carbonyls and assorted chromophores. These modifications include the homogenization of solid samples with high ionic strength buffers and treating the samples with acid and organic solvents to eliminate interfering chromophores such as haemoglobin, retinoids or unreacted DNPH [reviewed by Estevez et al. (2008) and Hellwig (2020)]. Besides the in-solution procedure, in-gel techniques based on western blotting and ELISA-type immunodetection by DNPH-tagged protein antibodies, can also be applied (Meyer et al. 2012; Augustyniak et al. 2015). Despite of recent optimization of the DNPH method for the assessment of protein carbonylation in food systems (Soglia et al. 2016) and other biological samples (Colombo et al. 2016; Bayarsaikhan et al. 2019), this procedure suffers from lack of specificity and has been recurrently criticized for providing an unreliable estimation of the real extent of the protein oxidative damage.

**Fig. 3:**
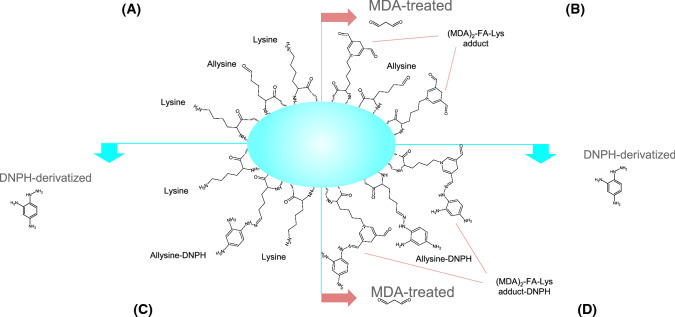
Illustration of the overestimation of primary carbonylation in malondialdehyde-exposed proteins by the dinitrophenylhydrazine method as reported by Estévez et al. (2019). *A-quadrant* occurrence of protein-bound lysine and *α*-AS residues, *B-quadrant* addition of malondialdehyde to protein-bound lysine residues, *C-quandrant* derivatization of protein-bound *α*-AS residues by the dinitrophenylhydrazine, *D-quadrant* derivatization of protein-bound α-AS and malondialdehyde residues by the dinitrophenylhydrazine

In regards to the specificity, it is generally recognized that the DNPH method provides limited information on the nature and or formation pathway of the quantified carbonyls (Hellwig 2020). Further to that, the overestimation of protein carbonyls by accounting lipid carbonyls introduced into proteins has been recently reported as a major flaw of the DNPH method (Estévez et al. 2019). The authors found that MDA readily reacts with ε-amino groups from protein-bound lysine residues and as a result, the DNP hydrazones quantified correspond to a major extent (up to 80% of total protein carbonyls) to protein-bound MDA carbonyl moieties. Therefore, at a certain range of MDA concentrations (0.05–1 mM), both the formation of primary protein carbonyls by either MCO or MR pathways, and their subsequent detection, is partially blocked by MDA and likely, other lipid-derived carbonyls. It is hence, worth taking into consideration that under severe lipid oxidative environment and MDA production, protein carbonyls quantified by DNPH may mostly reflect a secondary carbonylation process (Fig. 3).

To gain specificity and information of mechanistic nature, the analysis of specific protein carbonyls by chromatographic techniques, such as *α*-AS and γ-GS, is the election technique. These carbonyl compounds can be tagged by several derivatization procedures, eventually separated, and detected by HPLC or GC attached to fluorescence or MS detectors (Utrera et al. 2011; Estévez 2011; Hellwig 2020). *p*-Amino benzoic acid (*p*-ABA) has been used to tag and stabilize protein carbonyls prior to the hydrolysis procedure. The subsequent detection and quantification of *p*-ABA-derivatized carbonyls has been performed using MS (Estévez et al. 2009) and fluorescence detectors (Akagawa et al. 2009; Utrera et al. 2011). Alternative means of detection involves derivatization with fluoresceinamine (FINH_2_) prior to separation and identification using HPLC–MS (Climent et al. 1989). The analysis of protein carbonyls by GC involves the prior preparation of volatile hydroxyl derivatives from *α*-AS (hydroxyaminovaleric acid; HAVA) and *γ*-GS (hydroxyaminocaproic acid; HACA) and subsequent reduction with sodium borohydride (NaBH_4_) (Requena et al. 2001). Table 1 shows the concentration of protein carbonyls in various food products as analysed by the DNPH method or chromatographic techniques. Other innovative approaches include hydrazine-based derivatization prior to a 2D-electrophoresis gel analysis of redox proteomics (Malheiros 2019) and the cellular localization of protein carbonyls using mass spectrometry imaging (Flinders et al. 2015). The description of these and other methods for assessing protein oxidation in meat samples have been reviewed by Fedorova et al. (2014), Alomari et al. (2018), Hawkins and Davies (2019) and Hellwig (2020). Recent redox-proteomic approaches have contributed to understanding the occurrence of protein carbonylation and its underlying mechanisms in post-mortem muscle from mammals (Sayd et al. 2012), seafood (Lin et al. 2021), processed meat (Malheiros 2019; Mitra et al. 2018) and various biological samples (Verrastro et al. 2015; Driessen et al. 2015).

**Table 1:** Concentration of protein carbonyls in various food products reported in recent literature

Food system	Method	Protein carbonyls^A^	References
Raw lamb cutlets	DNPH	9	Lahmar et al. (2018)
Ready-to-eat chicken patties	DNPH	15	de Santana Neto et al. (2021)
Soy protein isolate	DNPH	13.6	Yu et al. (2018)
Soy protein isolate	DNPH	10.4	Zhang et al. (2017)
Milk	DNPH	23.6^B^	Kalaitsidis et al. (2021)
Yogurt	DNPH	11.8^B^	Kalaitsidis et al. (2021)
Feta cheese	DNPH	30.9^B^	Kalaitsidis et al. (2021)
Cooked ham	DNPH	8	Armenteros et al. (2016)
Rainbow trout mince	DNPH	3.6	Bitalebi et al. (2019)
Surimi fishballs	DNPH	4.2	Zhao et al. (2019)
Powdered infant milk	DNPH	3.1	Chen et al. (2019)
Silver carp fillets	DNPH	4	Zhang et al. (2021)
Raw shrimp	DNPH	11.9	Ruvalcaba-Márquez et al. (2021)
Blood meal	DNPH	127	Frame et al. (2020)
Raw rabbit meat	DNPH	7	Wang et al. (2018)
Air-dried yak meat	DNPH	8.5	Ma et al. (2021)
Fresh pork	DNPH	4.5	Hernández-López et al. (2016)
Cooked beef patties	HPLC^C^	1.3	Rysman et al. (2016)
Frozen rainbow trout fillet	HPLC^C^	4.5	Timm-Heinrich et al. (2013)
Fermented sausages	HPLC^C^	0.9	Öztürk‐Kerimoğlu et al. (2019)
Fermented sausages	HPLC^C^	0.9	Villaverde et al. (2014)
Jerky chicken	HPLC^C^	85	Silva et al. (2016)
Cooked bacon	HPLC^C^	80	Soladoye et al. (2017)
Sous vide-cooked lamb loin	HPLC^C^	0.42	Roldán et al. (2014)
Raw wooden chicken breast	HPLC^C^	3.2	de Carvalho et al. (2021)

*DNPH* quantification of protein carbonyls as hydrazones according to the dinitrophenylhydrazine method, *HPLC* quantification of specific protein carbonyls, α-AS and γ-GS, by high-performance liquid chromatography

^A^Results expressed as nmol of protein carbonyls/mg protein, unless otherwise noted

^B^Results expressed as ng of protein carbonyls/mL sample

^C^Results refer to the sum of *α*-AS and *γ*-GS

## Concise update on the biological consequences of protein carbonylation

Beyond their role as indicators of the extent of the oxidative damage to proteins, protein carbonyls may be directly implicated in the onset of processes leading to food quality deterioration, nutritional impairments, and pathophysiological conditions. The oxidation of lysine residues may have consequences on protein functionality and on the susceptibility of proteins to hydrolytic degradation. Further, protein carbonyls formed from lysine oxidation are reactive compounds, which are involved in the formation of crosslinks and carboxylic acids, with all of these having consequences of biological relevance. In this section, recent advances in the understanding of the impact of protein carbonylation on food quality, nutrition and health are concisely reported.

### Protein carbonylation and food protein functionality and digestibility

Proteins are major components of foods from animal origin, and, therefore, play an indisputable role in the technological, nutritional, and sensory properties of muscle, dairy and egg foods. Soon after the discovery that muscle proteins were susceptible to oxidation and protein carbonyls were quantified in food samples, this oxidative damage was linked to loss of protein functionality including impairments of the ability to form gels, emulsions and hold water (Xiong 2000). In subsequent studies, carbonylation was also associated to decreased susceptibility of oxidized proteins to proteases, which had consequences on meat tenderization during aging (Rowe et al. 2004) and on protein degradation during digestion of severely oxidized foods (Sante-Lhoutellier et al. 2007; Soladoye et al. 2015). In a previous comprehensive review about protein carbonylation in meat systems, Estévez (2011) already warned that most causative connections between protein carbonyls and the alleged effects on protein functionality were based on the calculations of positive and significant correlations. Since correlation does not involve causation, only by defining the underlying molecular mechanisms of the deleterious effects of protein carbonylation on protein functionality and food quality, the causative connection between both events can be rationally supported. In this regard, great efforts have been made in the last years to comprehend such connections. Utrera and Estévez (2012) thoroughly reported the occurrence of the “carbonylation pathway” in myofibrillar proteins subjected to a radical generating system. The formation of α-AS, as an early product from lysine oxidation, was followed by the formation of its oxidative degradation product (*α*-AA) and the formation of Schiff bases upon reaction of *α*-AS with protein-bound amino groups. These chemical changes were proposed to contribute to the impaired water functionality of meat proteins. Namely, the authors indicated that protein carbonylation could likely alter protein-water molecular interactions by (i) modification of the isoelectric point in oxidized proteins and (ii) by formation of protein aggregates; with both processes explaining an impaired protein functionality. Conversely, Bao et al. (2018) observed that protein carbonylation and protein crosslinks affected protein net charges though protein functionality was eventually improved. Yet, the authors induced oxidation by hypochlorous acid, and the results seemed to diverge from those reported by Utrera and Estévez (2012) who employed a hydroxyl radical oxidation system. Using a similar in vitro radical-induced oxidation system, Zhang et al. (2020) recently confirmed that protein crosslinking, aggregation and other severe structural changes in oxidized proteins compromise their water solubility and functionality. As recently reviewed by Zhao et al. (2021), the functionality of muscle proteins, including their ability to hold water and form gels, can be modulated by an appropriate management of the nature and extent of protein oxidation. Xiong (2000) already reported that mild and progressive protein oxidation may facilitate protein functionality and leads to stable gels, while severe oxidation leads to decreased protein functionality and impaired rheological properties. Numerous scientific evidences support this hypothesis and interestingly, phytochemicals (polyphenols, phenolic acids) may contribute to modify the functional and rheological properties of gels from mild to moderately oxidized proteins (Guo and Xiong 2021). The carbonylation of proteins via the “Suyama” pathway (Maillard-mediated mechanism) has also been found to affect the functionality of meat, dairy and soy proteins (Luna and Estévez 2019; Feng et al. 2021). Luna and Estévez (2019) observed that incubation of *β*-lactoglobulin and myofibrillar proteins with GO and MGO (2 M) at 80 °C cause severe carbonylation (> 20 nmo/mg protein) and reduced ability of the carbonylated proteins to hold water (90% loss of functionality). Glucose-treated soy proteins have also been reported to undergo extremely severe carbonylation and impaired surface properties when heated at 60 °C for 24 h (Feng et al. 2021). The occurrence of secondary carbonylation has also been found to affect protein functionality though the effect depends on the nature of the lipid-derived carbonyl and its concentration. Cheng et al. (2021a, b) recently observed that 5–20 mM MDA improved the WHC and rheological properties of gels from myofibrillar proteins while at even higher MDA concentrations (40 mM), the emulsion gel was completely lost. It is worth clarifying that these experimental MDA concentrations are out of the range found in processed or digested food products (Steppeler et al. 2016). Table 2 shows recent data on the concentration of protein carbonyls, MDA and 4-HNE in food digests. Wang et al. (2021) reported deleterious effects of 5 mM MDA on the functionality of myofibrillar proteins while these effects were found to be counteracted by phenolic terpenes such as linalool or limonene. The MDA-induced oxidative stress and consequent impaired functionality in myofibrillar proteins can also be modulated by the ionic strength of the media, which is dependent, in turn, on NaCl concentration (Zhou et al. 2015). In a recent paper, Keller et al. (2020) reported the heme–iron-induced production of 4-HNE in the intestinal lumen and the role of this reactive carbonyl in the formation of protein-adducts in heart, liver and skeletal muscle of rats.

**Table 2:** Concentration of protein carbonyls (PC), malondialdehyde (MDA) and 4-hydroxynonenal (4-HNE) in various food digests

Food system	GID^A^	PC^B^	MDA	4-HNE	References
Processed pork	Simulated static system (i)	8	30^C^	–	Van Hecke et al. (2018)
Mackerel	Simulated static system (i)	13	0.88^D^	34^E^	Van Hecke et al. (2019)
Salmon	Simulated static system (i)	9.5	0.53^D^	19^E^	Van Hecke et al. (2019)
Tuna	Simulated static system (i)	5.5	0.55^D^	13^E^	Van Hecke et al. (2019)
Parma ham	Simulated static system (g)	9	65^F^	8^F^	Goethals et al (2020)
Cooked chicken patties	Simulated static system (i)	10	124^F^	200^F^	Sobral et al. (2020)
Dry-cured loin	Simulated static system (i)	23	0.8^G^	–	Lavado et al. (2021)
Red cured cooked meat	Simulated static system (g)	8	0.4^D^	140^E^	Van Hecke et al. (2021)
	Sprague–Dawley rats (g)	7	100^F^	–	Van Hecke et al. (2021)
Cooked pork	Simulated static system (g)	5	5^H^	240^I^	Li et al. (2021a, b)

^A^*GID* Gastrointestinal digestion system. (i) data collected at the intestinal digestion phase. (g) data collected at the gastric digestion phase

^B^Results expressed as nmol of protein carbonyls/mg protein

^C^Results expressed as nmol/mL digest

^D^Results expressed as mmol/kg digest

^E^Results expressed as μmol/kg digest

^F^Results expressed as nmol/g digest

^G^Results expressed as μmol/mL digest

^H^Results expressed as μg/mL digest

^I^Results expressed as ng/mL digest

The impact of protein carbonylation on protein digestibility has also been a topic of recurring debate. As for the impact on the functional properties, the effect of the carbonylation of the susceptibility of proteins to undergo hydrolytic degradation depends on the severity of the oxidative damage. In this regard, mild protein oxidation may favour partial unfolding which facilitates enzymatic approach, recognition and action on the substrate. Conversely, the formation of irreversible protein aggregates owing to a severe and enduring oxidative damage may lead to a decreased proteolytic susceptibility (Soladoye et al. 2015). Morzel et al. (2006) ascribed the decreased susceptibility of oxidized myofibrillar proteins to papain, to the carbonylation of arginine and lysine as this enzyme hydrolyses at bonds involving such amino acids. In this regard, similar effects could be expected for digestive enzymes with a similar hydrolytic pattern like trypsin. Protein carbonyls could also impair the digestibility of food proteins by contributing to the formation of insoluble aggregates via carbonyl–amine crosslinks. Sante-Lhoutellier et al. (2007) found that the activity of trypsin/*α*-chymotrypsin on proteins decreased as the carbonylation level in the latter increased, which provides strength to the role of protein carbonylation on the impaired digestibility of oxidized proteins. According to recent literature, the carbonylation of proteins via the Maillard reaction (Luna and Estévez 2019; Wu et al. 2021) or via introduction of secondary lipid carbonyls such as MDA (Niu et al. 2019; Li et al. 2021a, b) have a deleterious impact on the digestibility of meat, dairy and cereal proteins. Recent studies have also established plausible connections between the extent of protein carbonylation and a reduced digestibility of proteins in a variety of foods such as egg white (Cheng et al. 2021a, b), whey isolate (Niu et al. 2019), ready-to-eat chicken patties (Ferreira et al. 2018), fish fillets (Semedo Tavares et al. 2018) and air-dried yak meat (Ma et al. 2021), among many others.

### Protein carbonylation and toxicological concerns

**Fig. 4:**
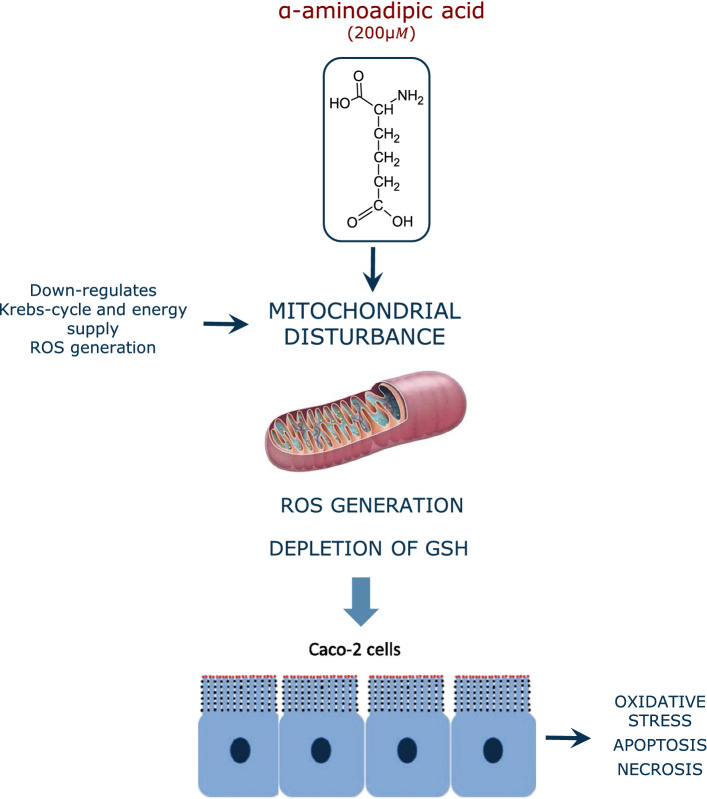
Mechanisms of the cytotoxic effects of α-AA on CACO-2 cells as reported by Díaz-Velasco et al. (2020)

The intake of oxidized lipids is known to increase the post-prandial plasma levels of oxidation and inflammation markers (Sies et al. 2005; Estévez et al. 2017). A sustained exposure to dietary lipid oxidation products is associated with an increased risk of suffering certain chronic inflammatory diseases and cancer (Sies et al. 2005). The molecular basis of this pathogenesis is well documented, as lipid-derived carbonyls such as MDA are known to display cytotoxic and mutagenic potential in the gastrointestinal tract (GIT) or in internal organs upon absorption (Esterbauer 1993). In this scenario, it is reasonable to enquiry whether dietary protein carbonyls may also contribute to impair cell homeostasis and induce health disorders. There is, in this regard, a profound lack of knowledge. The high reactivity of *α*-AS and other protein carbonyls, and the poor commercial availability of purified compounds, hinders the implementation of toxicological studies using such species. Alternatively, experimental animals have been fed with severely oxidized proteins in which carbonyls appeared to be one of the most salient chemical modifications. Using this experimental approach, Li et al. (2014) found that dietary oxidized proteins promoted the collapse of endogenous antioxidant cell defense systems, leading to the onset of oxidative stress and degeneration in kidney and liver of mice. The intake of severely carbonylated beef proteins to Wistar rats has been reported to have a significant impact on gut microbiota (Van-Hecke et al. 2021). To similar conclusions came Ge et al. (2020) who found that the alteration of the gut microbiota in mice by the intake of oxidized pork contributed to the onset of oxidative stress and inflammation processes in this murine model. The same authors reported that dietary oxidized pork induced impaired lipid metabolism by decreasing insulin levels and suppressing of the insulin receptor substrate-1 (IRS-1)/phosphoinositide 3-kinase (PI3K)/protein kinase B (Akt) signaling pathway and its downstream signaling molecules (Ge et al. 2021). Li et al. (2019) observed impaired spatial learning and memory in rats fed with carbonylated proteins from a severely processed milk product. While these studies support the role of dietary protein oxidation on physiological disorders, the identification of specific protein oxidation products as responsible for such effects is unfeasible. To comprehend the impact of particular oxidized amino acids, we recently carried out an experiment (unpublished data) in which Wistar rats were exposed to *α*-AS/piperideine-6-carboxylate in acute (5 mg/day/kg live weight for 4 weeks) and chronic assays (0.5 mg/day/kg live weight for 20 weeks). Among the most remarkable results, we observed the onset of oxidative stress in several tissues (e.g. small intestine, liver, spleen) and impaired lipid metabolism in treated rats vs. the control counterparts, which agrees with results obtained from some of the aforementioned studies. The most abundant protein carbonyl, *α*-AS, is oxidized in the presence of peroxides to the lysine oxidation end product, the *α*-AA. This transformation is readily taking place in pro-oxidative environments such as that created in hyperglycemic conditions (Luna et al. 2021). Unlike its precursor, this oxidized amino acid has been profusely studied in regards to its ability to induce pathological conditions. In a recent study, Díaz-Velasco et al. (2020) documented that the exposure of human CACO-2 cells to a food-relevant α-AA concentration (200 μM) leads to depletion of glutathione, oxidative stress, apoptosis and necrosis (Fig. 4). In a further study using proteomics (unpublished data), the *α*-AA was found to act as analog of glutamic acid and impair multiple biological processes in human intestinal cells such as the transepithelial transport, mitochondrial activity and protein repair mechanisms. Wang et al. (2013) induced pancreatic malfunction and diabetes in C57BL/6 mice by oral administration of α-AA (500 mg/day/kg live weight). Estaras et al. (2020) challenged mice pancreatic acinar cells with 200 μM *α*-AA and observed oxidative stress, calcium homeostasis dysfunction and impaired trypsin secretion. More details on the effect of dietary protein oxidation on human health can be found elsewhere (Estévez and Luna 2017; Estévez and Xiong 2019). Likewise, the application of antioxidant strategies to mitigate the negative consequences of the onset of lipid and protein oxidation in foods and in the gastrointestinal tract have been recently reviewed critically and comprehensively (Nieva-Echevarría et al. 2020; Lund 2021; Estévez 2021; Xiong and Guo 2021).

### Protein carbonylation as mechanism of cell communication

For a long time regarded as markers of protein oxidation, protein carbonyls have been found to participate actively in biological processes and metabolic functions. The understanding of the molecular basis of such bioactivities may be required to identify the role of protein carbonyls in the onset of physiological impairments and pathological conditions. Considering that protein carbonyls are still highly reactive and can be involved in carbonylamine reactions, with this reaction being key in many enzymatic and biological processes, it is plausible to hypothesize that protein carbonyls may act as signalling molecules. The role of protein carbonylation as a reaction of biological consequences is relevant from a food and nutrition perspective, considering the accretion of carbonyls in dietary proteins during processing, storage, culinary preparation and digestion. While the toxicological aspects of *α*-AS, as major protein carbonyl, and its oxidation product *(α*-AA), are still being examined, the dietary exposure to physiological concentrations of these oxidized amino acids may also play a role in the regulation of certain internal physiological processes upon absorption. Unfortunately, these effects are poorly understood. Likewise, the in vivo carbonylation of proteins in cells may also be implicated in mechanisms of cell regulation and control. In the following lines, the most recent discoveries of protein carbonyls as redox signaling molecules are concisely reviewed.

One of the main biological consequences of protein carbonylation is the activation of the protein turnover mechanism. As protein carbonylation is irreversible and cellular enzymes are unable to repair such oxidative damage, carbonylated proteins are degraded by the cell's proteasomal system (Baraibar and Friguet 2012). Otherwise, severely carbonylated proteins may not only be dysfunctional, they tend to form aggregates via increasing intrinsic protein hydrophobicity and the formation of carbonylamine reactions (Schiff-base structures) between protein carbonyls and *ε*-amino groups from protein-bound alkaline amino acids (i.e. lysine). The accumulation of aggregates of oxidized proteins in cells and tissues has been highlighted as a pathological hallmark of serious health disorders such as the Alzheimer disease (Levine 2002; Sharma et al. 2020). While cells may degrade aberrant proteins by whether the ATP-ubiquitin dependent 26S proteasome or by the ATP-ubiquitin independent 20S proteasome, carbonylated proteins seem to be preferentially degraded by the 20S proteasome (Jung and Grune 2008; Baraibar and Friguet 2012; Lefaki et al. 2017). It is worth noting that certain proteins may be more susceptible to carbonylation than others but the molecular basis of this specificity still remained unclear though it may respond to mechanisms of protein tagging in turn over or other cellular signaling mechanisms (Maisonneuve et al. 2009). Additionally, severely carbonylated and aggregated proteins are not recognized by the 20S proteasome, which leads to accretion of such oxidized complexes in cell.

Other works indicate the role of protein carbonylation on mitochondrial function and insulin resistance in muscle and adipose tissue (Frohnert and Bernlohr 2013). According to these authors, carbonylation of proteins may display opposite effects depending on the role of the proteins being affected by this post-translational modification. In this regard, certain proteins such as Kelch-like ECH-associated protein 1 (KEAP1) are activated in antioxidant response through carbonylation while insulin receptor substrates 1 and 2 are inactivated when carbonylated, which decreases insulin signaling. Another example is the activation of *c*-Jun N-terminal kinase (JNK) upstream kinase ASK1 by secondary carbonylation via 4-HNE adduction. The activation of this pathway leads to the ultimate expression of pro-inflammatory target genes, linking oxidative stress and inflammation (Curtis et al. 2012; Hecker and Wagner 2018). Proposing a pioneering theory, Wong et al. (2013) reported that primary protein carbonylation could be a physiological mechanism of ROS signaling and that such mechanism is regulated by decarbonylation. Therefore, according to their proposal, the formation of protein carbonyls could be reversed by the involvement of cell reductases, which clearly diverge from the well-established assumption that carbonylation was an irreversible damage to proteins. As a supportive example of their theory, the authors observed that carbonylation of annexin A1, which shows anti-inflammatory, anti-proliferative and pro-apoptotic effects, leads to its degradation by the proteasome. This oxidation-driven degradation of annexin A1 is proposed as a cell signal for cell growth. Though the authors suggest that certain enzymes and redox proteins, such as alcohol dehydrogenases and thioredoxin, may be involved in decarbonylation of proteins, it is currently unclear whether free or protein-bound oxidized amino acids such as *α*-AS or *γ*-GS could be reduced under physiological conditions.

## Concluding remarks

The recent advances achieved in the field of protein carbonylation facilitate the understanding of the biological significance of this multifaceted reaction. While the carbonylation on food proteins is typically regarded as a reaction of negative consequences owing to the loss of nutritional value, decreased digestibility and impaired functionality, scientific evidences support the benefits of mild and controlled oxidation of proteins in the improvement of their gel abilities and rheological properties. Likewise, the onset of protein carbonylation in cells may play a double role, depending on the severity of the reaction and the protein target of such post-translational modification. On the one hand, severe and enduring oxidative stress leading to massive protein carbonylation and aggregation may block protein turnover and cause pathological conditions related to oxidative stress and inflammation. On the other hand, mild and/or targeted carbonylation in particular proteins may induce physiological responses for the strengthening of the antioxidant defences, the activation of the protein turnover and likely modulation of the immune system. As a truly open topic, further and inspiring discoveries will contribute to consolidate the connections between protein carbonylation, food quality, nutrition and health.

## Abbreviations

4-HNE: 4-Hydroxynonenal
*α*-AA: *α*-Aminoadipic acid
*α*-AS: *α*-Aminoadipic semialdehyde
*γ*-GS: *γ*-Glutamic semialdehyde
AA: Acetaldehyde
DNPH: Dinitrophenylhydrazine
FINH_2_: Fluoresceinamine
GC: Gas chromatography
GIT: Gastrointestinal tract
GO: Glyoxal
HAVA: Hydroxyaminovaleric acid
HACA: Hydroxyaminocaproic acid
HPLC: High-performance liquid chromatography
HSA: Human serum albumin
MCO: Metal-catalysed oxidation
MDA: Malondialdehyde
MGO: Methyl glyoxal
MR: Maillard reaction
MS: Mass spectrometry
p-ABA: P-aminobenzoic acid
ROS: Reactive oxygen species
WHC: Water-holding capacity
